# Efficacy, safety, and post-vaccination antibody titer data in children with CAPS treated with Canakinumab

**DOI:** 10.1186/1546-0096-13-S1-P1

**Published:** 2015-09-28

**Authors:** P Brogan, M Hofer, J Kuemmerle-Deschner, B Lauwerys, A Speziale, K Abrams, K Leon, X Wei, R Laxer

**Affiliations:** 1 UCL Institute of Child Health, and Great Ormond Street Hospital NHS Foundation Trust, Department of Paediatric Rheumatology, London, UK; 2 Unité romande de rhumatologie pédiatrique, Hospitalier Universitaire Vaudois, Lausanne, Switzerland; 3 University Hospital Tuebingen, Tuebingen, Germany; 4 Cliniques Universitaires Saint-Luc and Université Catholique de Louvain, Brussels, Belgium; 5 Novartis Pharma AG, Basel, Switzerland; 6 Novartis Pharmaceuticals Corporation, New Jersey, USA; 7 Novartis Pharma, Beijing, China; 8 University of Toronto, Staff Rheumatologist, The Hospital for Sick Children, Toronto, Ontario, Canada

## Background

Canakinumab (CAN) is indicated for the treatment of cryopyrin-associated periodic syndrome (CAPS) in patients ≥2 years of age.[^1^] However, patients may require treatment in infancy where CAN has not yet been studied. IL-1 inhibition has not affected antibody production after vaccination in healthy volunteers[^2^], but no data in patients receiving standard childhood vaccines are available.

## Objectives

To evaluate the efficacy and safety of CAN, including post-vaccination antibody production, in children with CAPS <4 years of age.

## Methods

CAN-naïve patients aged 28 days to 4 years with CAPS received open-label CAN dosed 2-12 mg/kg every 4 or 8 weeks for 56 weeks. Efficacy was evaluated by complete response (clinical response and normal C-reactive protein [CRP]) and subsequent relapse. Safety was assessed by adverse event (AE) reporting and vaccination response evaluated by post-vaccine antibody titers measured at 28 and 57 days post vaccination. Vaccines evaluated included DTP; H. Flu; N. Men.; influenza; Hep B; and Strep. Pneum.

## Results

Of 17 patients enrolled, 6 were less than 24 months old (44 days-5 months). The phenotypic distribution was: FCAS (n=1), MWS (n=12), and NOMID (n=4). All 17 patients achieved a clinical response and 16 achieved a complete response. Seven patients required dose escalation to achieve and/or maintain their responses. The patient who did not achieve a complete response was a 1 year old with persistently elevated CRP. Of the 16 patients with a complete response, 4 (2 with MWS and 2 with NOMID) subsequently relapsed, but all regained complete response; 2 (1 MWS; 1 NOMID) with and 2 (1 MWS; 1 NOMID) without dose escalation. No CAPS flares were reported with vaccination and a rise in post-vaccination antibody titers was observed for all vaccines evaluated. The most common type of AE reported was an infection, typically involving the upper respiratory tract. Four patients experienced a serious AE (SAE), with no SAE occurring more than once. No patient discontinued due to an AE.

## Conclusions

Canakinumab is an effective treatment for patients with CAPS aged as young as 44 days old. Canakinumab appears to have no effect on the ability to produce antibodies against standard childhood non-live vaccines. The safety profile of canakinumab was acceptable and similar to that observed for older patients.
